# Specific Dephosphorylation at Tyr-554 of Git1 by Ptprz Promotes Its Association with Paxillin and Hic-5

**DOI:** 10.1371/journal.pone.0119361

**Published:** 2015-03-05

**Authors:** Akihiro Fujikawa, Masahito Matsumoto, Kazuya Kuboyama, Ryoko Suzuki, Masaharu Noda

**Affiliations:** 1 Division of Molecular Neurobiology, National Institute for Basic Biology, Okazaki, Aichi, Japan; 2 School of Life Science, The Graduate University for Advanced Studies, Okazaki, Aichi, Japan; Seoul National University, KOREA, REPUBLIC OF

## Abstract

G protein-coupled receptor kinase-interactor 1 (Git1) is involved in cell motility control by serving as an adaptor that links signaling proteins such as Pix and PAK to focal adhesion proteins. We previously demonstrated that Git1 was a multiply tyrosine-phosphorylated protein, its primary phosphorylation site was Tyr-554 in the vicinity of the focal adhesion targeting-homology (FAH) domain, and this site was selectively dephosphorylated by protein tyrosine phosphatase receptor type Z (Ptprz). In the present study, we showed that Tyr-554 phosphorylation reduced the association of Git1 with the FAH-domain-binding proteins, paxillin and Hic-5, based on immunoprecipitation experiments using the Tyr-554 mutants of Git1. The Tyr-554 phosphorylation of Git1 was higher, and its binding to paxillin was consistently lower in the brains of *Ptprz*-deficient mice than in those of wild-type mice. We then investigated the role of Tyr-554 phosphorylation in cell motility control using three different methods: random cell motility, wound healing, and Boyden chamber assays. The shRNA-mediated knockdown of endogenous Git1 impaired cell motility in A7r5 smooth muscle cells. The motility defect was rescued by the exogenous expression of wild-type Git1 and a Git1 mutant, which only retained Tyr-554 among the multiple potential tyrosine phosphorylation sites, but not by the Tyr-554 phosphorylation-defective or phosphorylation-state mimic Git1 mutant. Our results suggested that cyclic phosphorylation-dephosphorylation at Tyr-554 of Git1 was crucial for dynamic interactions between Git1 and paxillin/Hic-5 in order to ensure coordinated cell motility.

## Introduction

Git1, which is also referred to as Cool-associated, tyrosine-phosphorylated 1 (Cat-1), is a member of the GTPase-activating protein for ADP-ribosylation factor (Arf GAP) family [1]. It consists of an Arf GAP domain that inactivates Arf6 at the amino (N)-terminus [2, 3], ankyrin repeats, a Spa2-homology domain (SHD) [4], and a focal adhesion targeting-homology (FAH) domain at the carboxy (C)-terminus [5, 6]. The SHD was previously shown to be important for forming stable molecular complexes with the p21-activated kinases (PAKs) interacting exchange factor (Pix) family of Rho guanine nucleotide exchange factors [4, 7–10]. Git1-Pix complexes are known to be recruited to focal complexes through an interaction between the FAH domain of Git1 and paxillin [5, 6, 8], which promotes membrane protrusions at the leading edge of migrating cells [4, 8]. Git1 is a highly phosphorylated protein [11], and 13 potential tyrosine phosphorylation sites have been listed on the PhosphoSitePlus database (www.phosphosite.org). Although Git1 is tyrosine-phosphorylated in a Src-dependent manner [7, 12, 13], the involvement of respective tyrosine phosphorylation in Git1 in its binding to focal adhesion proteins has not yet been elucidated in detail. The replacement of 10 tyrosine residues of potential phosphorylation in Git1 with a phenylalanine residue was previously shown to result in almost the complete disappearance of its tyrosine phosphorylation in HEK293T cells, even under the condition of the expression of v-Src or treatment with pervanadate [12]. Git1 was recently found to be preferentially phosphorylated at Tyr-554, moderately at Tyr-392 and Tyr-607, and faintly at Tyr-519 [12].

Reversible protein tyrosine phosphorylation controlled by protein-tyrosine kinases (PTKs) and protein-tyrosine phosphatases (PTPs) is critical for the regulation of numerous cellular events including cell adhesion and migration [14]. PTP receptor type Z (Ptprz, also called PTPζ or RPTPβ), a receptor-like PTP (RPTP) belonging to the R5 subfamily together with Ptprg (PTPγ), is expressed as a chondroitin sulfate proteoglycan in the brain [15, 16]. Ptprz has been detected on the rims of growth cones and filopodial processes in cultured cortical neurons [17], in which actin-rich structures play important roles for axonal path finding and neurite outgrowth. A number of studies using various cell species have indicated that Ptprz is involved in regulating cellular attachment and migration by binding with multiple extracellular binding molecules including heparin-binding growth factors, such as pleiotrophin [17, 18] and midkine [19], and the glycosylphosphatidylinositol (GPI)-anchored molecule, F3 [20].

As substrate molecules that transduce signals downstream of Ptprz, we previously identified several cellular proteins including Git1 by developing a genetic method named “the yeast substrate-trapping system” to screen for PTP substrates [13, 21]. The substrate site was subsequently revealed in Git1 at Tyr-554 and also in the GTPase-activating protein for Rho GTPase (p190RhoGAP) at Tyr-1105, membrane-associated guanylate kinase, WW and PDZ domain-containing 1 (Magi1) at Tyr-373, and paxillin at Tyr-118 [12]. We consequently elucidated that the typical substrate motif for Ptprz was Glu/Asp-Glu/Asp-Glu/Asp-Xaa-Ile/Val-Tyr(P)-Xaa (Xaa is not an acidic residue) [12].

The physiological importance of Ptprz has been demonstrated through studies using *Ptprz*-deficient mice, in which a maturation-dependent enhancement in long-term potentiation in the CA1 region of the hippocampus along with learning deficits [22–24], alterations in oligodendrocyte differentiation and myelination in the central nervous system [25, 26], and reductions in sensorimotor responses [27] were reported. Tyrosine phosphorylation at Tyr-1105 of p190RhoGAP was found to be decreased during contextual fear conditioning in the hippocampus of wild-type mice, but not in *Ptprz*-deficient mice [22]. Git1 is also expressed in hippocampal neurons and localizes to synapses [28, 29], and *Git1*-knockout mice were previously reported to exhibit deficits in the acquisition of conditioned fear [30] and impairments in the formation of dendritic spines in the hippocampus [31]. In peripheral tissues, gastric mucosal cells express a nonproteoglycan form of Ptprz, albeit at lower levels [32]. *Ptprz*-deficient mice were shown to be resistant to gastric ulceration by VacA, a cytotoxin secreted by *Helicobacter pylori* [32]. The tyrosine phosphorylation level of Git1 was increased in cultured gastric cells treated with VacA, indicating that Ptprz functions as a receptor of VacA for gastric mucosal damage [32].

Among the multiple phosphorylation sites in Git1 by Src, Ptprz preferentially dephosphorylated phospho-Tyr-554 [12]. We assumed that cyclic phosphorylation-dephosphorylation at Tyr-554 by Src and Ptprz was involved in an important function of Git1. Therefore, we herein investigated the role of Tyr-554 phosphorylation in Git1, with a particular focus on molecular interactions with other molecules and its cellular functions. We revealed that the Tyr-554 phosphorylation of Git1 weakened its association with the FAH-domain-binding proteins, paxillin and Hic-5. Furthermore, we found that the ability of Git1 to promote cell motility was impaired by both phosphorylation-defective and phosphorylation-mimic mutations at Tyr-554 of Git1.

## Materials and Methods

### Antibodies

The following are the specificities and sources of antibodies used: Against phosphotyrosine (PY20; GE Healthcare), the FLAG epitope (mouse monoclonal M2, F3165, and rabbit anti-FLAG, F7425; Sigma), the Myc epitope (rabbit anti-Myc, 600–401–381; Rockland, and mouse monoclonal 9E10; Sigma), GFP (mouse monoclonal anti-GFP, 11–814–460–001; Roche), Hic-5 (mouse monoclonal anti-Hic-5, 611164; BD Biosciences), paxillin (mouse monoclonal anti-paxillin, 610569; BD Biosciences, and rabbit anti-paxillin, sc-5574; Santa Cruz Biotechnology), and Git1 (rabbit anti-Git1, sc-13961; Santa Cruz Biotechnology, and mouse anti-Git1 monoclonal antibody, 611396; BD Biosciences). Rabbit antisera specific for the amino acid residues 251–555 of Git1 (anti-GIT1/Cat-1) [13], rabbit polyclonal antibodies against phospho-Tyr-554 on Git1 (anti-pY554-Git1) [12], and a rabbit anti-Ptprz-S serum [15] were prepared in our laboratory. Anti-pY554-Git1 antibodies were conjugated with horseradish peroxidase (HRP) using a peroxidase labeling kit (Dojindo Molecular Technologies).

### Mammalian expression plasmids and shRNAs

The plasmid series of pFLAG-Git1 were used for the expression of FLAG-tagged Git1 and its tyrosine mutants [12]. The plasmid series of pFLAG-mCherry-Git1 for the expression of mCherry (red fluorescent protein)-fused Git1 proteins were generated by the in-frame insertion of mCherry cDNA (GenBank accession no. AY678264) into the pFLAG-Git1 series (between the N-terminal FLAG epitope and Git1 ORF). pYFP-Git1 for YFP (yellow fluorescent protein)-fused Git1 was generated by the in-frame insertion of the EYFP cDNA of the pEYFP-C1 vector (Clontech) into pcDNAGIT1 [11]. The other expression constructs of the Myc-tagged proteins, pMyc-Hic-5, pMyc-βPix, and pMyc-paxillin, were generated by inserting their full-length cDNAs (mouse Hic-5, BC056362; mouse βPix, NM_001113517; and mouse paxillin, AF293882) into the pcDNA-Myc vector [21]. cDNAs were obtained by RT-PCR from mouse brain total RNA. Mission shRNA vectors including the Git1-specific shRNA vector (pLKO.1-Git1, NM_001004144) and control vector (pLKO.1, SHC002) were purchased from Sigma.

### Cell culture and DNA transfection

HEK293T cells (human embryonic kidney epithelial cells) were maintained on dishes coated with rat tail collagen in Dulbecco’s modified Eagle’s medium (DMEM) supplemented with 10% fetal bovine serum (FBS) in a humidified incubator at 37°C with 5% CO_2_. The DNA transfection of HEK293T cells was performed using the standard calcium phosphate technique [12]. A7r5 (rat aorta smooth muscle) cells were purchased from DS Pharma Biomedical and maintained in DMEM supplemented with 10% FBS. The DNA transfection of A7r5 cells or its stable transformants was performed using Lipofectamine 2000 (Life Technologies). Transfected cells were cultured for 24 h, and replated into appropriate dishes. After the stable knockdown of *Git1* in A7r5 cells was performed with the vector pLKO.1-Git1, cells were selected with puromycin (5 μg/ml).

### Immunoprecipitation experiments

In order to screen the molecules that bind to Git1, HEK293T cells transfected with the indicated plasmids were treated with 100 μM pervanadate for 15 min. Cells were extracted with lysis buffer, 20 mM Tris-HCl, pH 7.4, 1% NP-40, 150 mM NaCl, 10 mM NaF, 1 mM sodium orthovanadate, and an EDTA-free protease inhibitor cocktail (complete EDTA-free, Roche), and then centrifuged at 15,000 *g* for 15 min. The supernatants were mixed with anti-FLAG M2 magnetic beads (Sigma) by rotation for 3 h. After washing the beads, the bound proteins were eluted with FLAG elution solution (Sigma) according to the manufacturer’s instructions. In the co-immunoprecipitaion assays, the bound proteins were eluted by boiling with SDS-PAGE sample buffer and subjected to Western blotting.

To analyze tyrosine phosphorylation *in vivo*, 4-month-old *Ptprz*-deficient [23] and wild-type C57BL/6 male mice were habituated for at least 2 h to a new homecage and then decapitated. The cerebral cortex was rapidly dissected out on ice, weighed, and immediately used for the preparation of crude synaptosomes as described [33]. The synaptosome extracts, which were pretreated with protein G Sepharose (GE Healthcare) for 30 min at 4°C, were incubated with rabbit anti-GIT1/Cat-1 antisera-coated Protein-G Sepharose for 1 h at 4°C to examine Git1 phosphorylation at Tyr-554. The same extracts were incubated with mouse monoclonal anti-paxillin-coated beads to examine the endogenous interaction of Git1 with paxillin. The immunocomplexes were eluted by boiling with SDS-PAGE sample buffer. All animal experiments were performed according to the guidelines for Animal Care with approval by the Committee for Animal Research, National Institutes of Natural Sciences.

### Western blotting

Samples were subjected to SDS-PAGE followed by semi-dry electroblotting onto a polyvinylidene difluoride (PVDF) membrane (Immobilon-P, Millipore). The membrane was incubated for 1 h in a blocking solution (4% non-fat dry milk and 0.1% Triton X-100 in 10 mM Tris-HCl, pH 7.4, 150 mM NaCl), and incubated overnight with the respective antibodies in the blocking solution. The binding of these antibodies was detected with an ECL Western blotting system (GE Healthcare). In order to detect phosphorylated proteins, the membranes were blocked with 1% BSA and 0.1% Triton X-100 in 10 mM Tris-HCl, pH 7.4, 150 mM NaCl, and then incubated with the HRP-conjugated anti-phospho-Tyr mAb (clone PY20) or rabbit anti-pY554-Git1 antibodies.

### Live-cell imaging and random cell motility assay

A7r5 cells and its transfectants were replated on fibronectin (50 μg/ml)-coated glass-bottomed dishes in OPTI-MEM (Life Technologies) supplemented with 2% FBS. Live-cell imaging was performed using a microscope system equipped with a temperature- and CO_2_-controlled chamber (Biostation IM, Nikon). The expression of mCherry-Git1 was verified using an appropriate filter set (excitation wavelength at 540/25 nm; emission wavelength at 605/55 nm) before initiating image capturing. Phase contrast (PhC) images were taken with a 40x objective lens every 5 min for 3 h (for Wind-Rose plots), or every 10 sec for 30 min (for kymographs) per experiment. The paths of cell movement were traced by following nuclei between frames of the movies, and the trajectory length from the traced images was measured with Adobe Photoshop CS6 (Adobe). Kymographs were generated using ImageJ (rsb.info.nih.gov) installed with multiple kymograph plugins. The resulting image represented membrane activity (y axis) over time (x axis). The protrusion frequency was defined as the number of peaks extending >0.5 μm.

### Boyden chamber and wound healing assays

Parental A7r5 and *Git1*-knockdown cells transfected with mCherry or mCherry-fused Git1 constructs were cultured for 48 h, and mCherry-positive cells were sorted by flow cytometry (Cell Sorter SH800, Sony). Boyden chamber assays were performed using 24-well transwell inserts with an 8-μm pore-sized membrane (Chemotaxicell, Kurabo) and 24-well culture plates (BD Falcon). Cells in 250 μl of OPTI-MEM containing 100 μg/ml BSA were added to a fibronectin (50 μg/ml)-coated transwell insert (1.5 × 10^5^ cells per well). The lower chambers were filled with 200 μl of the medium containing epidermal growth factor (EGF, 15 ng/ml) as a chemoattractant. After a 6-h incubation, cells fixed with 10% neutral formalin were stained with DAPI (4′, 6-diamidino-2-phenylindole). Cells present in the upper chamber were removed with cotton swabs, and cells that migrated to the lower surface were counted using a conventional fluorescence microscope. In the wound healing assay, cells (1.0 × 10^5^ cells per well) were replated on fibronectin (50 μg/ml)-coated 24-well plastic plates (BD Falcon), and then cultured with DMEM supplemented with 10% FBS until monolayers formed. After changing the medium to OPTI-MEM containing 100 μg/ml BSA and 10 ng/ml EGF, cell monolayers were scratched with a sterile micropipette tip, and then allowed to migrate for 48 h.

## Results

### Identification of molecules that interact differently with Git1 by phosphorylation at Tyr-554

**Fig 1:**
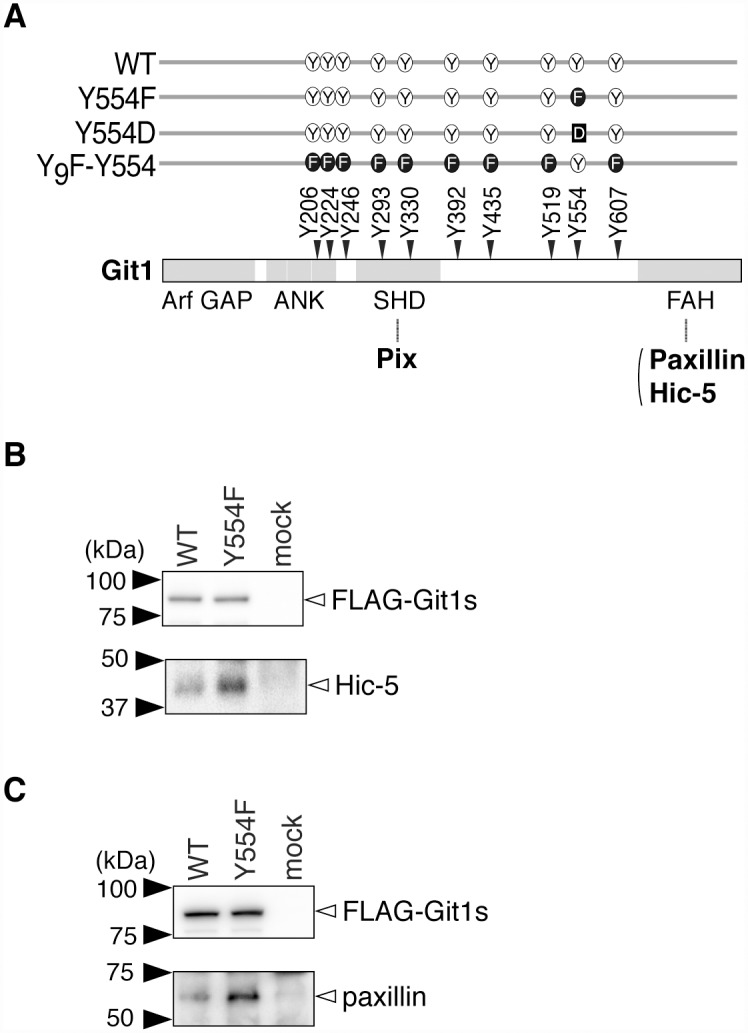
Hic-5 was more abundant in immunoprecipitates of the Git1-Y554F mutant than in those of WT. ***A***, Schematic representation of Git1. The Git1 mutant constructs used in this study are shown with their abbreviated names. We previously showed that the replacement of ten potential phosphorylation tyrosine residues of Git1 with a phenylalanine residue resulted in the almost total disappearance of its tyrosine phosphorylation in cells, even after the pervanadate treatment, and then constructed Y_9_F-Y554 from the decuple mutant by changing Phe-554 back to tyrosine [12]. Pix binds to the SHD [4, 7–10], and Hic-5 and paxillin to the FAH [5, 6, 8]. Arf GAP, the GTPase-activating protein (GAP) domain for ADP-ribosylation factor (Arf); ANK, ankyrin repeats; SHD, Spa2 homology domain, FAH; focal adhesion targeting (FAT) homology domain. Y, tyrosine; F, phenylalanine; D, aspartic acid. ***B***, ***C***, Immunoprecipitation experiments. HEK293T cells expressing FLAG-tagged Git1 (WT), FLAG-tagged Git1-Y554F (Y554F), or a control vector (mock) were treated with 100 μM pervanadate for 15 min. The cell extracts were incubated with anti-FLAG beads, and the binding proteins were then specifically eluted with FLAG peptides. The eluates were separated by SDS-PAGE, followed by Western blotting with a rabbit anti-FLAG, mouse anti-Hic-5 (B), or anti-paxillin antibody (C).

Git1 was preferentially phosphorylated at Tyr-554, which was located between the SHD and FAH domains (see Fig. 1A). To examine the functional roles of Tyr-554 phosphorylation in Git1, we prepared FLAG-tagged expression constructs for wild-type Git1 (WT) and its Tyr-554 phosphorylation-defective mutant (Y554F), and transfected them into HEK293T cells. After the tyrosine phosphorylation of cellular proteins was induced with the membrane-permeable PTP inhibitor, pervanadate, we performed immunoprecipitation experiments with anti-FLAG beads from the cell extracts. In the immunoprecipitate of Y554F, several bands with sizes ranging between 40 and 60 kDa were observed by CBB staining of the SDS gel, in addition to those in the immunoprecipitate of WT transfection. Of these, a 40-kDa band that reproducibly gave an intense signal in the immunoprecipitate of Y554F was identified as Hic-5 by Western blotting (Fig. 1B); see also our previous paper [12] in which this was discussed as an unpublished observation. A 60-kDa band was concomitantly identified as paxillin (Fig. 1C).

### Decreases in the binding activity of Git1 to Hic-5 and paxillin by Tyr-554 phosphorylation

Hic-5 and its family member, paxillin, contain LD (leucine-aspartate repeat) motifs at the N-terminus and LIM (lin11, isl-1, and mec-3) domains at the C-terminus [34]. Previous studies reported that LD3 in Hic-5 and LD4 in paxillin directly bound to the FAH domain of Git1 [5, 6]; however, the tyrosine phosphorylation-dependent regulation mechanism of Git1 binding to paxillin or Hic-5 has yet to be elucidated in detail, including the responsible tyrosine phosphorylation site in Git1 [5, 35].

**Fig 2:**
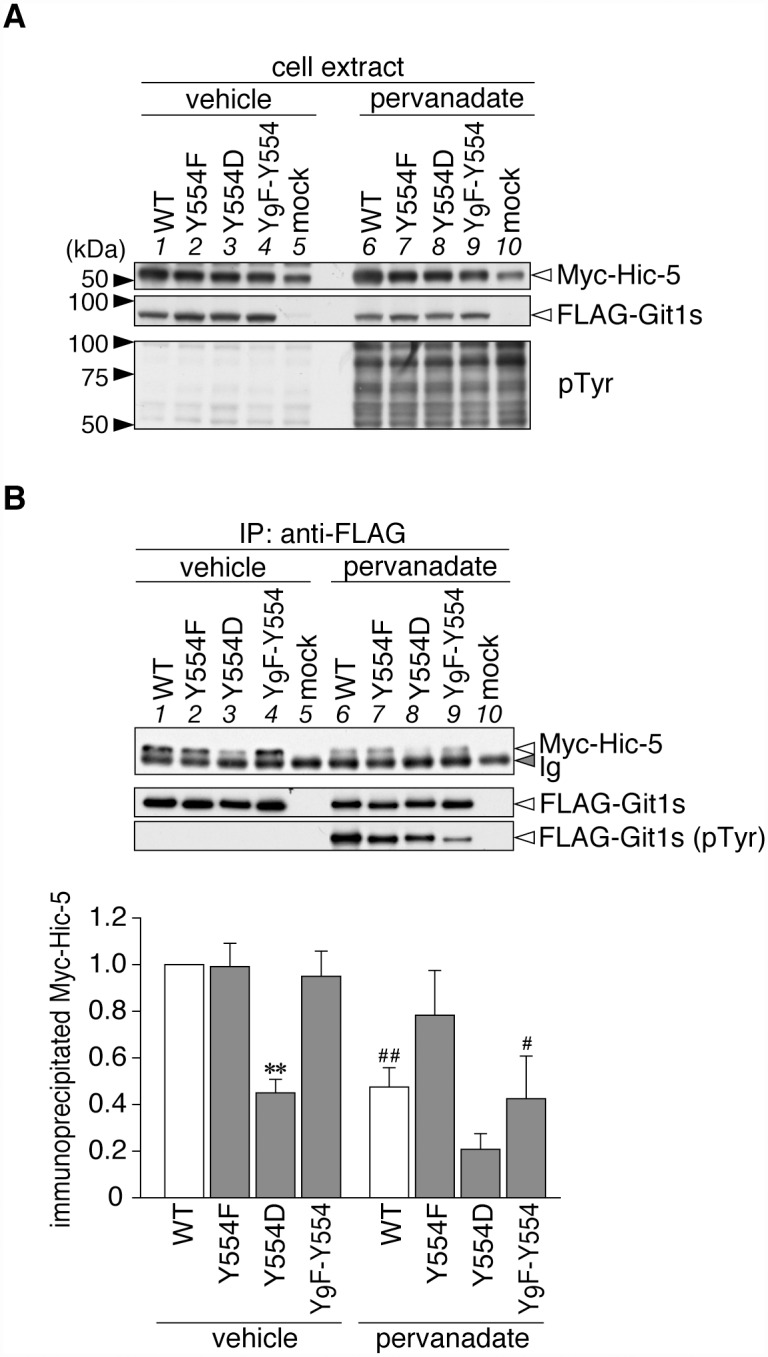
Git1 phosphorylation at Tyr-554 weakened its association with Hic-5. ***A***, Western blotting of protein expression levels, and tyrosine phosphorylation of all proteins in HEK293T cells expressing FLAG-tagged Git1 proteins (Fig. 1A) together with Myc-tagged Hic-5. Cells were treated with 100 μM pervanadate or vehicle for 15 min, and then analyzed by Western blotting using anti-FLAG M2, anti-Myc 9E10, or anti-phosphotyrosine PY20. ***B***, Co-immunoprecipitaion of Git1 mutants with Hic-5. The immunoprecipitates from cell extracts with anti-FLAG beads were analyzed by Western blotting with an anti-FLAG or anti-Myc antibody. To verify the tyrosine phosphorylation of FLAG-tagged Git1 proteins, the same membrane was reacted with anti-phosphotyrosine PY20. Ig, immunoglobulin. The lower part shows the densitometric analysis of the relative amount of Myc-Hic-5 to FLAG-Git1 in the immunoprecipitates. Data are the mean ± S.E. (error bars; *n* = 3). **, *P* < 0.01 significantly different from the wild-type with the same treatment; ^#^, *P* < 0.05 or ^##^, *P* < 0.01 significant difference between vehicle- and pervanadate-treated groups by ANOVA with Fisher’s PLSD *post hoc* tests.

To determine the effects of Tyr-554 phosphorylation on the binding of Git1 to Hic-5 and paxillin, we exogenously expressed FLAG-tagged wild-type or mutant Git1 in HEK293T cells, together with Myc-tagged Hic-5. We verified the appropriate protein expression of transfected molecules and intensive tyrosine phosphorylation of cellular proteins by the pervanadate treatment (Fig. 2A). The robust tyrosine phosphorylation of FLAG-tagged Git1 proteins in the anti-FLAG immunoprecipitates was confirmed (Fig. 2B), as described previously [12]. Western blot analyses of immunoprecipitates from cell extracts with anti-FLAG demonstrated that WT and Y554F showed robust Hic-5-binding activities under basal conditions without pervanadate (Fig. 2B; compare lanes 1 and 2), whereas the binding activity of the phosphorylation-state mimic Tyr(554)Asp mutant (Y554D) was markedly weaker than that of WT (lanes 1 and 3). As expected from the results of Fig. 1B, the binding of WT to Hic-5 was significantly decreased by the pervanadate treatment (lanes 1 and 6), while the binding capacity of Y554F remained unchanged (lanes 2 and 7). The binding activity of Y554D was slightly reduced by the pervanadate treatment (lanes 3 and 8), suggesting that Tyr-554 may be the primary, but not sole phosphorylation site for the negative regulation of the association between Git1 and Hic-5. As was expected, Y_9_F-Y554 showed decreased binding to Hic-5 with the pervanadate treatment as well as WT (lanes 4 and 9): Y_9_F-Y554 is only phosphorylated at Tyr-554; see also [12]. Thus, the single-site phosphorylation at Tyr-554 was revealed to be sufficient to weaken Git1 binding to Hic-5.

**Fig 3:**
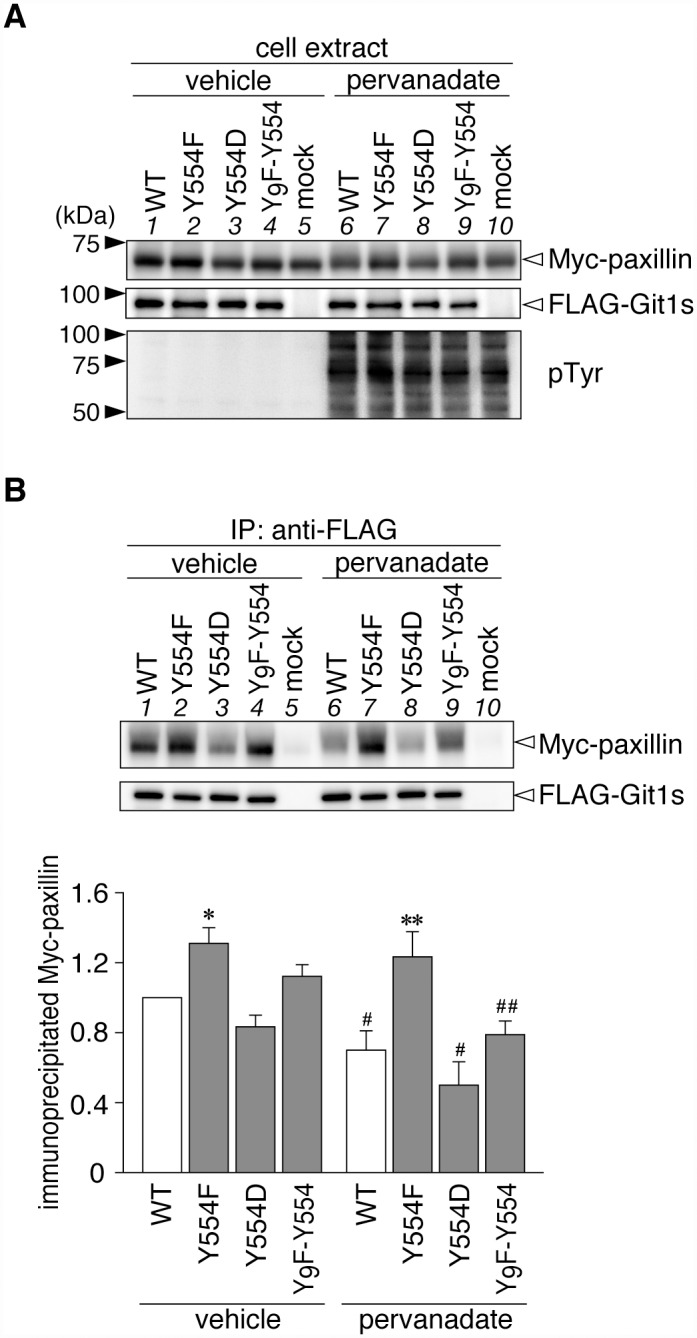
Git1 phosphorylation at Tyr-554 weakened its association with paxillin. ***A***, Western blotting of protein expression levels, and tyrosine phosphorylation of all proteins in HEK293T cells expressing FLAG-tagged Git1 proteins together with Myc-tagged paxillin. Western blotting analyses were performed using anti-FLAG M2, anti-Myc 9E10, or anti-phosphotyrosine PY20. ***B***, Co-immunoprecipitaion of Git1 mutants with paxillin. The amount of FLAG-Git1 and Myc-paxillin in the anti-FLAG immunoprecipitates was determined as above. Data are the mean ± S.E. (error bars; *n* = 3). *, *P* < 0.05 or **, *P* < 0.01 significantly different from the wild-type with the same treatment; ^#^, *P* < 0.05 or ^##^, *P* < 0.01 significant difference between vehicle- and pervanadate-treated groups by ANOVA with Fisher’s PLSD *post hoc* tests.

**Fig 4:**
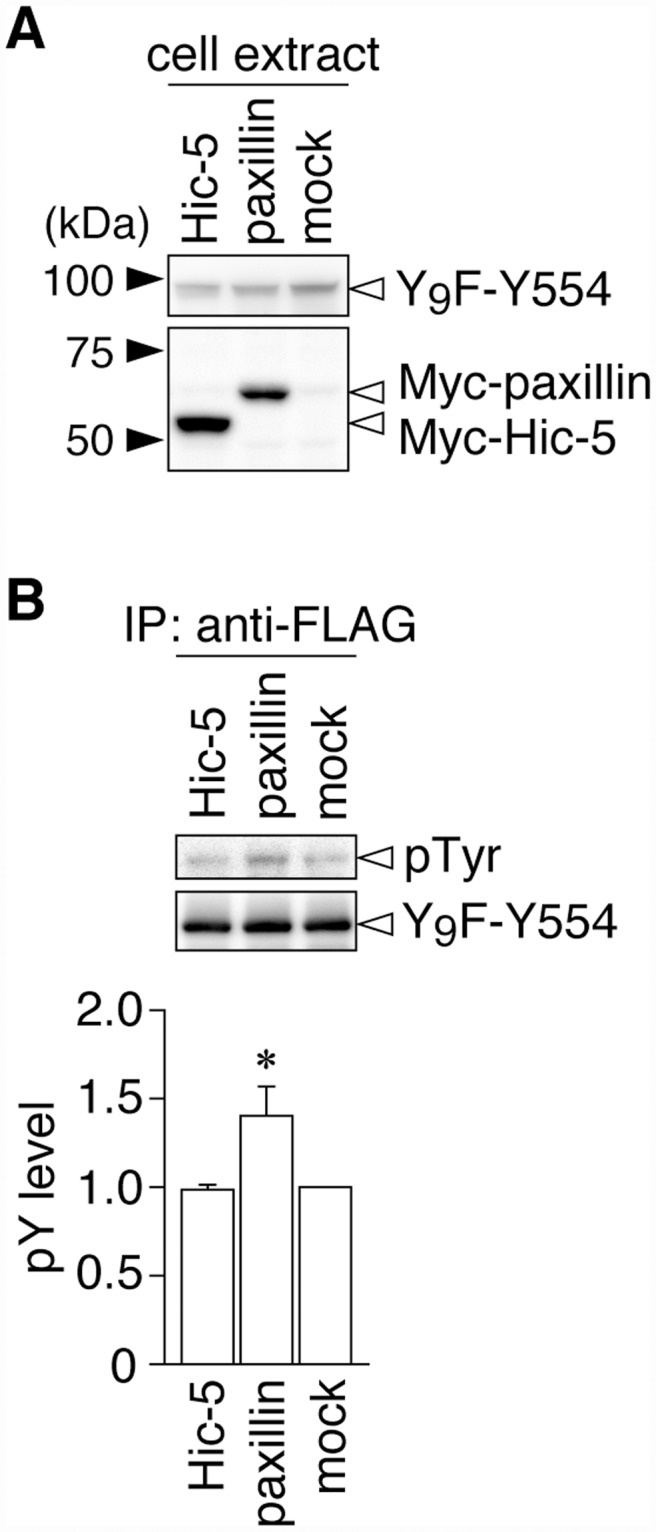
Git1 phosphorylation at Tyr-554 was enhanced by co-expression of paxillin. ***A***, Western blotting of protein expression levels in HEK293T cells exogenously expressing the FLAG-tagged Git1-Y_9_F-Y554 mutant together with Myc-tagged paxillin, Myc-tagged Hic-5, or a control mock. ***B***, Tyrosine phosphorylation of Y_9_F-Git1 proteins in anti-FLAG immunoprecipitates. The lower graph shows the densitometric analysis of the Western blotting data. Data are the mean ± S.E. (error bars; *n* = 3). *, *P* < 0.05 significantly different from Hic-5-transfected cells by ANOVA with Fisher’s PLSD *post hoc* tests.

As shown in Fig. 3, Git1 binding to paxillin was also reduced by Tyr-554 phosphorylation, similar to Hic-5. Following the pervanadate treatment, binding ability to paxillin was only maintained in Y554F (lane 2 to lane 7), which was the same as that for Hic-5 (see Fig. 2). The following differences were noted; the binding of Y554F to paxillin (lane 2) was significantly higher than that of WT (lane 1), while the binding of Y554D (lane 3) was similar to that of WT under basal conditions. These differences under basal conditions were attributed to greater tyrosine phosphorylation at Tyr-554 of Git1 by the co-expression of paxillin, than that of Hic-5 (Fig. 4). Thus, Tyr-554 phosphorylation appeared to weaken the Git1 association with both Hic-5 and paxillin even though Tyr-554 was located away from the FAH domain in the primary structure.

**Fig 5:**
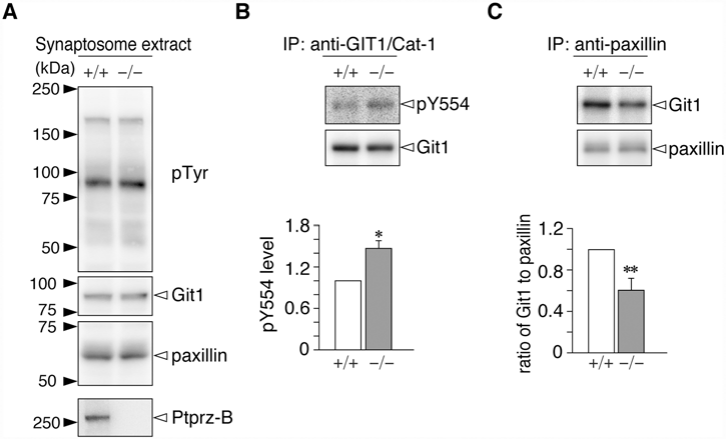
Comparison of Git1 phosphorylation at Tyr-554 and its binding to paxillin between wild-type and *Ptprz*-deficient mice. ***A***, Western blotting of cerebral synaptosome extracts prepared from the brains of wild-type (+/+) and *Ptprz*-deficient (−/−) mice. The overall tyrosine phosphorylation pattern and protein expression were analyzed with an anti-phosphotyrosine PY20 antibody, and rabbit anti-Git1 and anti-paxillin antibodies, and rabbit anti-Ptprz-S, respectively. The Ptprz-B isoform was previously shown to be detectable after the chondroitinase-ABC treatment [16] because Ptprz proteins are expressed as chondroitin-sulfate proteoglycans in the brain [15]. ***B***, Tyr-554 phosphorylation of Git1. The synaptosome extracts were immunoprecipitated with rabbit anti-GIT1/Cat-1 antisera-coated beads, and the binding proteins were analyzed by Western blotting with rabbit anti-pY554-Git1 and mouse anti-Git1 antibodies. The Tyr-554 phosphorylation level was determined by densitometric analyses. Data are the mean ± S.E. (error bars; *n* = 5). *, *P* < 0.05 significantly different from the wild-type by the Student’s *t*-test. ***C***, Git1 binding to paxillin. The immunoprecipitated proteins with mouse anti-paxillin were analyzed by Western blotting with rabbit anti-Git1 and rabbit anti-paxillin antibodies. The amount of Git1 relative to that of paxillin was determined by densitometric analyses. Data are the mean ± S.E. (error bars; *n* = 6). **, *P* < 0.01 significantly different from the wild-type by the Student’s *t*-test.

Since Ptprz [15], Git1 [2], and paxillin [8] are preferentially expressed in the brain, we attempted to compare endogenous phosphorylation levels at Tyr-554 of Git1 in the brains of wild-type and *Ptprz*-deficient mice [23], as well as its binding to paxillin. No genotypic differences in the overall tyrosine phosphorylation pattern or expression levels of Git1 and paxillin were observed in the cerebral synaptosome fractions of the neocortex (Fig. 5A), in which the Ptprz-B isoform was expectedly detected in the wild-type only: see also [16, 33]. The Tyr-554 phosphorylation of Git1 was significantly higher in *Ptprz*-deficient mice than in wild-type mice (Fig. 5B), and the amount of Git1 that co-immunoprecipitated with paxillin was accordingly decreased (Fig. 5C). Therefore, Ptprz appears to regulate interaction dynamics between Git1 and paxillin in the brain through the dephosphorylation of phospho-Tyr-554: see also [12].

### No effects of the Tyr-554 phosphorylation of Git1 on its binding to Pix or homo-oligomerization

**Fig 6:**
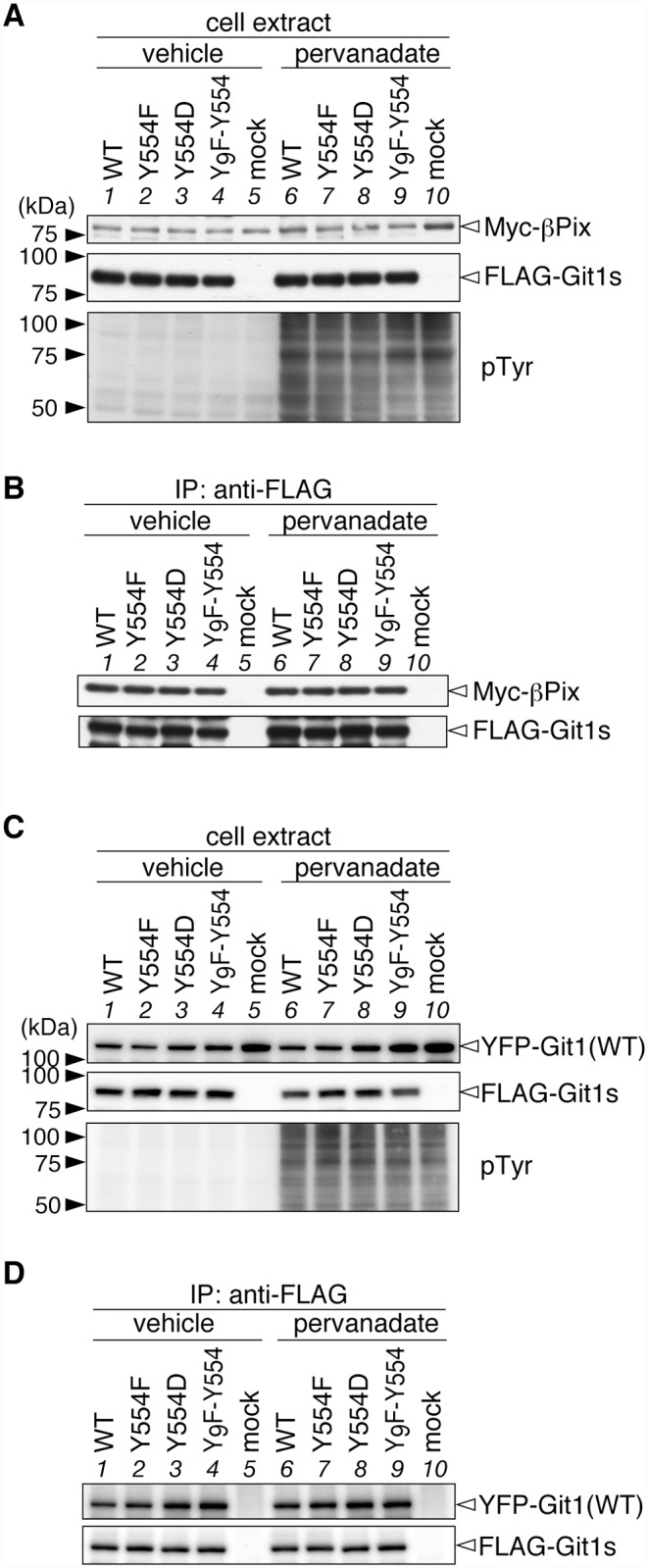
Tyr-554 phosphorylation of Git1 did not affect its binding to Pix or self-oligomerization. ***A***, Western blotting of protein expression levels, and tyrosine phosphorylation of all proteins in HEK293T cells expressing FLAG-tagged Git1 proteins together with Myc-tagged Pix. ***B***, Co-immunoprecipitaion of Git1 mutants with βPix using anti-FLAG beads. The figures are representative of two separate experiments. ***C***, Western blotting of HEK293T cells expressing FLAG-tagged Git1 mutants together with YFP-fused wild-type Git1. FLAG-tagged and YFP-tagged Git1 proteins were detected with anti-FLAG M2 and anti-GFP antibodies, respectively. ***D***, Co-immunoprecipitaion experiments using anti-FLAG beads. The figures are representative of two separate experiments.

Git1 has been shown to form stable complexes with the Rac/Cdc42-specific exchanging factor, Pix through interactions between the SHD domain of Git1 and the Git1 binding domain of Pix [9, 10]. Since Tyr-554 is located between the SHD and FAH domains of Git1, we determined whether Tyr-554 phosphorylation affects Git1-Pix complex formation by the exogenous co-expression of FLAG-tagged Git1 mutants with Myc-tagged βPix. We showed that the amount of co-immunoprecipitated βPix with wild-type Git1 was the same as that with the Tyr-554 Git1 mutants (Figs. 6A and 6B, lanes 1 to 5). Furthermore, the pervanadate treatment did not affect the Git1-βPix association (Figs. 6A and 6B, lanes 6 to 10). Thus, Tyr-554 phosphorylation weakened Git1 binding to Hic-5 and paxillin without affecting Git1-Pix complex formation.

Since Tyr-554 is located relatively close to the region (amino acid residues 428–485) of Git1 that forms a coiled-coil structure and mediates homodimerization [9, 10], we also examined the effects of Tyr-554 phosphorylation on the homophilic interactions of wild-type Git1 (YFP-tagged) by its co-expression with Git1 mutants (FLAG-tagged) in various combinations. Anti-FLAG immunoprecipitation experiments using the cell extracts demonstrated that the amount of co-immunoprecipitated Git1 proteins did not differ among the combinations with different mutants (Figs. 6C and 6D). This result indicated that the phosphorylation state at Tyr-554 did not affect the homodimerization of Git1.

### Effects of the Git1 Tyr-554 mutation on cell motility and lamellipodial dynamics in A7r5 cells

**Fig 7:**
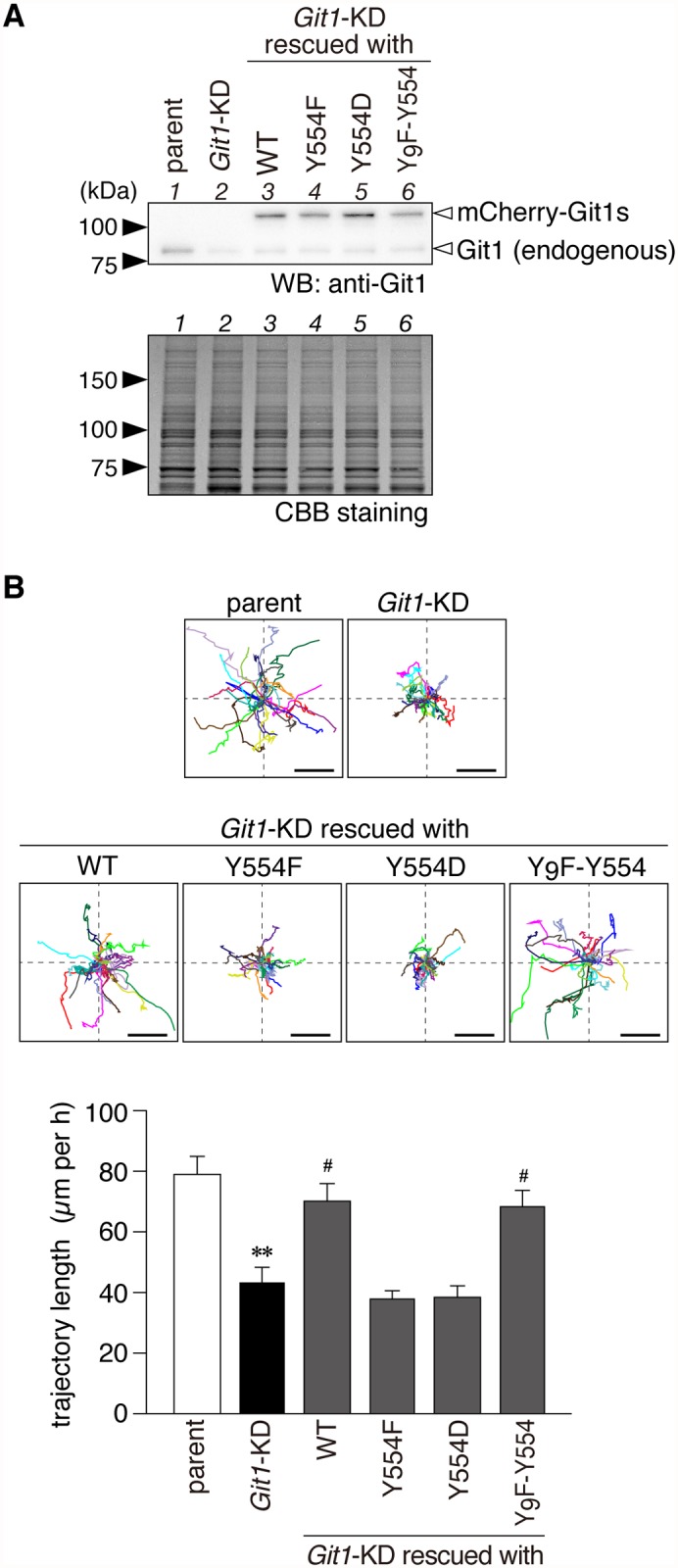
Git1 Tyr-554 mutants failed to restore impaired cell motility by *Git1* knockdown in a random cell motility assay. ***A***, Western blotting of Git1 expression in parental A7r5 cells, *Git1*-knockdown (*Git1*-KD) cells, and *Git1*-KD cells exogenously expressing the mCherry-fused Git1 mutants (WT, Y554F, Y554D, and Y_9_F-Y554). Both the endogenous Git1 and mCherry-fused Git1 mutant proteins were detected with anti-Git1. The protein amounts applied were verified by CBB staining. ***B***, Wind-Rose plots of individual cell trajectories on fibronectin-coated dishes. Live-cell images were taken every 5 min for 3 h per experiment. Before beginning the recording, the expression of mCherry-fused proteins was verified in the rescue experiments by a short exposure to UV to avoid possible cell damage. Each wind-rose plot showed tracks from a total of 23 cells per group, with the initial position of each track being superimposed at a common origin for clarity. Scale bars, 100 μm. The lower graph shows the length along the trajectory. Data are the mean ± S.E. (error bars; *n* = 23). **, *P* < 0.01 significantly different from parental cells; ^#^, *P* < 0.05 significantly different from *Git1*-KD cells by ANOVA and Scheffé’s *post hoc* tests.

Git1 has been shown to play an important role in cell motility by controlling lamellipodial dynamics [4, 5, 35–41]. To examine the effects of Tyr-554 phosphorylation on cellular behaviors, we first established a stable host cell line of A7r5 rat smooth muscle cells, which were devoid of endogenous Git1 due to the forced expression of a specific shRNA targeting the 3’-UTR of rat *Git1* mRNA. Western blotting showed that the amount of endogenous Git1 was less than 20% in knockdown (*Git1*-KD) cells as compared with that in parental cells (Fig. 7A, lane 1 to lane 2). Live-cell imaging of single cells demonstrated that random motility was markedly lower in *Git1*-KD cells than in parental cells (Fig. 7B).

**Fig 8:**
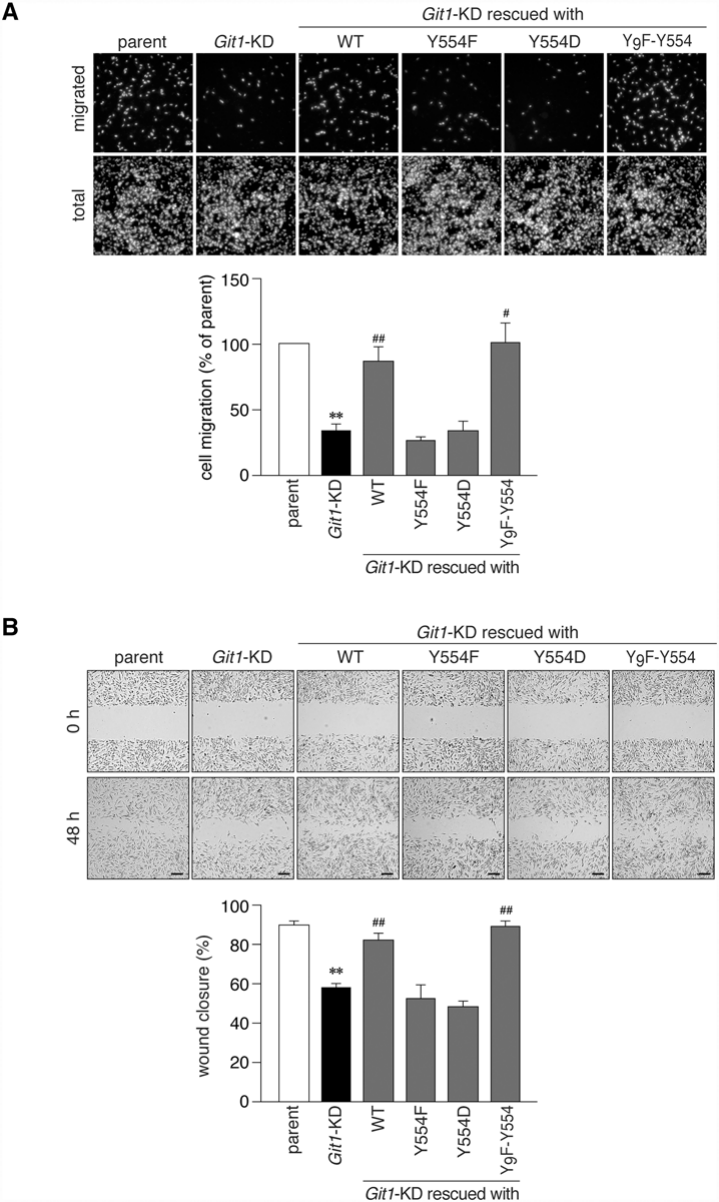
Git1 Tyr-554 mutants failed to restore cell mobility impaired by *Git1* knockdown in Boyden chamber and wound healing assays. ***A***, The Boyden chamber assay. Parental A7r5 cells or *Git1*-KD cells transfected with an mCherry or mCherry-fused Git1 construct were plated on the upper compartment of the fibronectin-coated chamber, and allowed to migrate to the lower side of the filter for 6 h. DAPI-stained nuclei were counted before (total) and after (migrated) the removal of cells remaining in the top chamber with cotton swabs. The lower graph shows the quantification of the migrated cells to the lower side. Data are the mean ± S.E. (error bars; *n* = 5). **, *P* < 0.01 significantly different from parental cells; ^#^, *P* < 0.05 or ^##^, *P* < 0.01 significantly different from *Git1*-KD cells by ANOVA and Scheffé’s *post hoc* tests. ***B***, The wound healing assay. Scrape wounds were introduced to the confluent monolayers of cells and then allowed to heal for 48 h. Scale bars, 200 μm. Wound areas were measured at 0 and 48 h. The lower graph shows the percentage of wound closure 48 h after the original wound. The mean ± S.E. (error bars; *n* = 7). **, *P* < 0.01 significantly different from parental cells; ^##^, *P* < 0.01 significantly different from *Git1*-KD cells by ANOVA and Scheffé’s *post hoc* tests.

We then performed rescue experiments by transfecting expression constructs coding mCherry-fused Git1 mutants, but lacking the 3’-UTR sequence of rat *Git1*; therefore, the mRNAs were insensitive to *Git1* shRNA. The amounts of plasmids used in these experiments were carefully adjusted to attain the same expression level of Git1 as the endogenous level in parental cells (Fig. 7A). The reduced cell motility of *Git1*-KD cells was expectedly rescued to the level of parental cells by the restorative expression of mCherry-Git1 (WT) (Fig. 7B), indicating that the defect in cell motility was due to the loss of Git1 expression. We then transfected *Git1*-KD cells with the Tyr-554 mutants of Git1 (Fig. 7A, lanes 4 to 6). WT and Y_9_F-Y554 fully rescued the defect in motility, whereas neither Y554F nor Y554D did (Fig. 7B). Similar results were obtained in different assays; EGF-induced migration in the Boyden chamber (Fig. 8A) and cell monolayer repair in the wound healing assay (Fig. 8B). These results suggested that cyclic phosphorylation-dephosphorylation at Tyr-554 of Git1 may be required for co-ordinated cell movement.

**Fig 9:**
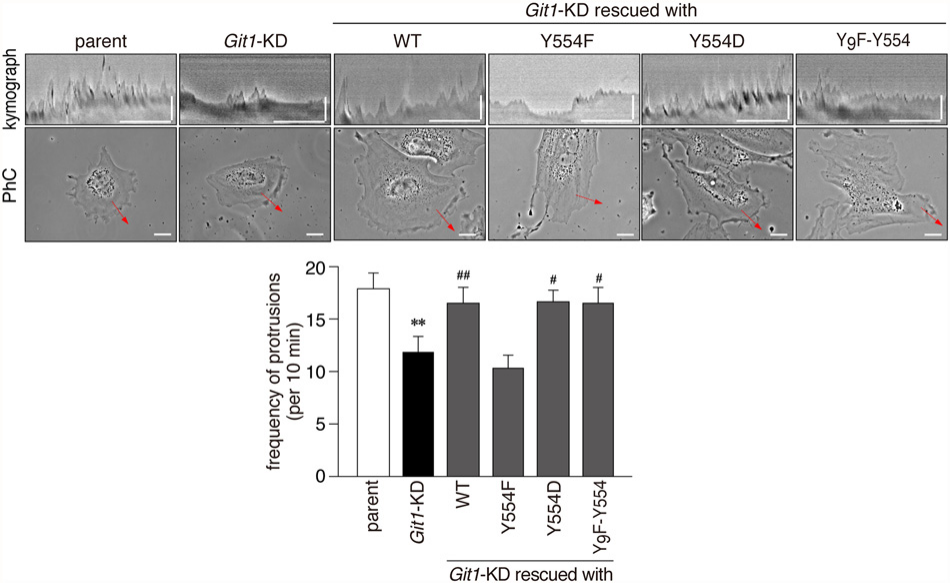
The phosphorylation-defective Tyr-554 mutant of Git1 failed to restore impaired lamellipodial protrusion activity in A7r5 cells by *Git1*-knockdown. Representative kymographs (upper pictures; scale bars, 10 min on the x-axis and 10 μm on the y-axis) obtained from phase-contrast photographs (lower pictures; scale bars, 20 μm) of parental cells, *Git1*-KD cells, and *Git1*-KD cells expressing various mCherry-fused Git1 proteins on fibronectin-coated dishes. Before beginning the recording, the expression of mCherry-fused proteins was verified in the rescue experiments by a short exposure to UV to avoid possible cell damage. The lower graphs show the frequency of protrusions. Data are the mean ± S.E. (error bars; *n* = 10 to 11 each). **, *P* < 0.01 significantly different from parental cells; ^#^, *P* < 0.05 or ^##^, *P* < 0.01 significantly different from *Git1*-KD cells by ANOVA and Scheffé’s *post hoc* tests.

The kymograph analysis indicated that lamellipodial protrusion activity of *Git1*-KD cells was defective in contrast to parental cells (Fig. 9). The expression constructs of not only WT and Y_9_F-Y554, but also Y554D rescued lamellipodial protrusion activity in *Git1*-KD cells, whereas that of Y554F did not (Fig. 9), suggesting that the capability of phosphorylation at Tyr-554 was essential for Git1-mediated protrusion activity.

## Discussion

**Fig 10:**
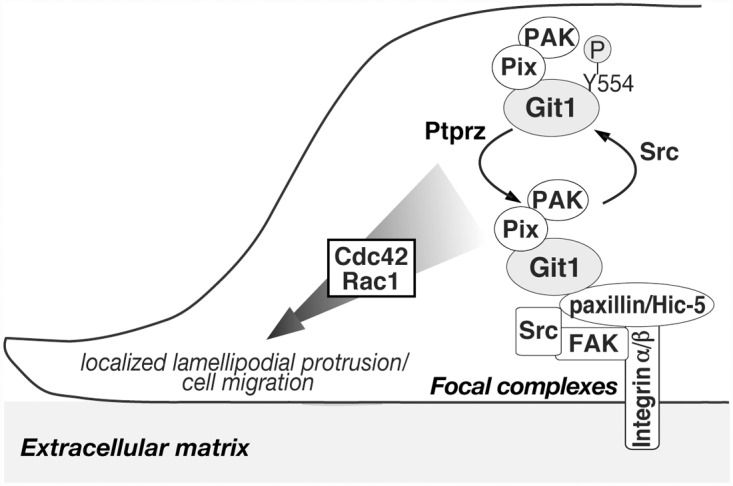
A proposed model. The Tyr-554 phosphorylation of Git1 is dependent on a Src tyrosine kinase [7, 12, 13]. The Git1-Pix complex dissociates from focal complexes by Tyr-554 phosphorylation, allowing the complex to recycle following dephosphorylation by tyrosine phosphatases, such as Ptprz [12, 13, 32]. Although FAK (focal adhesion kinase) is activated in response to integrin engagement, it is not yet clear whether FAK directly phosphorylates the Tyr-554 site. A stable complex of Git1-Pix containing PAK may reversibly associate with paxillin or Hic-5 at focal complexes by cyclic phosphorylation-dephosphorylation at Tyr-554, which is required to ensure the appropriate activation of Cdc42/Rac1 for localized protrusion activity in the front of the cells and co-ordinated cell movement: see also [4, 7–10, 35–41]. Here it should be noted that paxillin at Tyr-118 was also dephosphorylated by Ptprz [12]; however, the corresponding phosphorylation site is not present in Hic-5. The phosphorylated Tyr-118 and Tyr-31 residues reportedly serve as the binding sites for several src homology 2 (SH2) domain-containing proteins such as p120RasGAP and CrkII, and the phosphorylation of the two sites is necessary for efficient leading-edge protrusions during cell migration [42].

One important functional role of Git1 is to link the complex of PAK and Pix to remodeling focal adhesion proteins through interactions with paxillin or Hic-5, which may facilitate cell spreading and migration by enhancing focal adhesion turnover [4, 5, 35–41]. In the present study, we revealed that the Tyr-554 phosphorylation of Git1 attenuated its association with paxillin and Hic-5 without affecting Git1-Pix complex formation. Neither the phosphorylation-defective nor phosphorylation-state mimic Tyr-554 mutant of Git1 rescued cell motility impaired by the knockdown of endogenous *Git1* expression. Our results indicated that the phosphorylation capability of Tyr-554 in Git1 was essential for normal migration, and suggested that cyclic phosphorylation-dephosphorylation at Tyr-554 in Git1 regulated the dynamic interaction between the Git1-Pix complex and the focal adhesion proteins, paxillin and Hic-5, to ensure co-ordinated cell migration (Fig. 10).

Git1 has been shown to bind with paxillin via the FAH domain (amino acid residues 647 to 770) of Git1, and the FAH-deleted Git1 mutant no longer binds to paxillin [5]. The phosphorylation of Ser-709 in this domain has been reported to regulate paxillin binding [43]. However, tyrosine phosphorylation within the FAH domain is unlikely to be involved in binding to paxillin [5], and has not yet been identified [11]. On the other hand, Tyr-554 of Git1 is located between the SHD (264 to 374) and FAH domains, therefore, it is unlikely that Tyr-554 itself directly constitutes a binding site for paxillin and Hic-5. As an attractive mechanism to regulate the Git1-paxillin interaction, de Curtis and co-workers proposed the existence of an equilibrium between the two conformation states of Git1 [35, 40]; a closed conformation in which the N-terminal portion of Git1 may interact with the C-terminal part to maintain the protein in a binding-incompetent state, and an open conformation that allows binding to paxillin. This group very recently reported that Tyr-246 and Tyr-293 residues located in the ANK repeats and SHD domain, respectively, were requisite for maintaining the closed conformation; however, their phosphorylation did not release the closed conformation [35]. They also showed that Src- and pervanadate-induced tyrosine phosphorylation in the full-length Git1 caused a moderate reduction in binding to paxillin [35]. Their finding is consistent with our results in Figs. 3 and 4. The Tyr-554 phosphorylation may somehow stabilize the closed conformation, and, thus, attenuate the binding of Git1 to both paxillin and Hic-5.

The binding capacity of the phosphorylation-state mimic Y554D to Hic-5 and paxillin was further reduced by pervanadate (Figs. 2 and 3). These results strongly suggested the presence of other phosphorylation sites that may synergistically assist in reducing FAH domain-mediated interactions. The findings of a systematic mass spectrometry-based study on Git1 appear to support this proposal [11]; most tyrosine phosphorylation sites were crowded (five out of seven sites in total; Tyr-392, Tyr-519, Tyr-554, Tyr-563, and Tyr-607) in the flanking region (amino acids 375–646) between the SHD and FAH domains of Git1. Of these, Premont and colleagues noted that the Y563F Git1 mutant enhanced the interaction with paxillin [5]. Although phosphorylated Tyr-392 reportedly acts as the docking site for the SH2 domain of the adaptor protein Grb4 [44], the functional roles of the three other sites remain unknown. The FAH-mediated binding of Git1 may be negatively regulated by sequential phosphorylation at Tyr-554 as the primary site and its surrounding Tyr-519, Tyr-563, and Tyr-607 as additive sites in a graded manner.

The knockdown of *Git1* resulted in impairments in cell migration activity (Figs. 7 and 8) as well as lamellipodial protrusion activity (Fig. 9) in A7r5 cells, as has also been reported in CHO-K1 cells [36]. The finding that a Git1 mutant lacking the FAH domain failed to mediate cell migration and protrusions [41] prompted us to presume that the attenuation of FAH domain-mediated binding activity by phosphorylation at Tyr-554 may also have affected cell migration ability by Git1. Neither the Y554F nor Y554D mutant rescued the defect in the motility of *Git1*-KD cells, in contrast to wild-type or Y_9_F-Y554 (Figs. 7 and 8). The expression of not only WT and Y_9_F-Y554, but also Y554D rescued lamellipodial protrusion activity in *Git1*-KD cells, whereas Y554F did not (Fig. 9). Therefore, the Tyr-554 phosphorylation of Git1 may be necessary and sufficient for cellular protrusion; however, the ability of the constitutively phosphorylated form to make cells migrate appears to be deficient.

These results are reminiscent of the finding that Git1 has a similar mechanism to that observed in vinculin, one of the core focal adhesion proteins [45]. Vinculin adopts either an extended conformation (active form) that binds many partners, or a closed conformation (inactive form) that masks the binding sites, and the cycles of the active-inactive forms of vinculin control focal adhesion dynamics and signaling to co-ordinate polarized cell motility [45]. Neither of the vinculin mutants mimicking the constitutively active or inactive form enhanced cell migration in B16F10 melanoma cells [45]. We propose a conceptually analogous model for Git1 in which cyclic phosphorylation-dephosphorylation at Tyr-554 of Git1 may be crucial for its dynamic interaction with the focal adhesion-associated proteins, paxillin and Hic-5, in order to ensure the appropriate activation of Cdc42/Rac1 for localized protrusion to the front and coordinated movement of the cell (Fig. 10).

In conclusion, we herein revealed the functional significance of Tyr-554 phosphorylation in Git1, the primary phosphorylation site by Src. The specific dephosphorylation at Tyr-554 by Ptprz appears to fine tune the targeting of Git1-Pix complexes to appropriate subcellular locations (Fig. 10). We consider this mechanism to be deeply implicated in multiple cellular events, such as adhesion, migration, differentiation, and synaptic formation.
